# Synthesis of Polyaniline Supported CdS/CdS-ZnS/CdS-TiO_2_ Nanocomposite for Efficient Photocatalytic Applications

**DOI:** 10.3390/nano12081355

**Published:** 2022-04-14

**Authors:** Nida Qutub, Preeti Singh, Suhail Sabir, Khalid Umar, Suresh Sagadevan, Won-Chun Oh

**Affiliations:** 1 Department of Chemistry, Jamia Millia Islamia, New Delhi 110025, India; 2 Department of Fibers and Textile Processing Technology, Institute of Chemical Technology, Mumbai 400019, India; aries.pre84@gmail.com; 3 Department of Chemistry, Aligarh Muslim University, Aligarh 202002, India; drsuhailsabir@gmail.com; 4 School of Chemical Sciences, Universiti Sains Malaysia (USM), Penang 11800, Malaysia; khalidumar4@gmail.com; 5 Nanotechnology & Catalysis Research Centre, University of Malaya, Kuala Lumpur 50603, Malaysia; 6 Department of Advanced Materials Science and Engineering, Hanseo University, Seosan-si 356-706, Chungnam, Korea

**Keywords:** nanoparticles, conducting polymer, Acid blue-29 dye, photocatalytic mechanism

## Abstract

Photocatalytic degradation can be increased by improving photo-generated electrons and broadening the region of light absorption through conductive polymers. In that view, we have synthesized Polyaniline (PANI) with CdS, CdS-ZnS, and CdS-TiO_2_ nanocomposites using the chemical precipitation method, characterized and verified for the photo-degradation of Acid blue-29 dye. This paper provides a methodical conception about in what way conductive polymers “PANI” enhances the performance rate of composite photocatalysts (CdS, CdS-ZnS and CdS-TiO_2_). The nanocomposites charge transfer, molar ratio, surface morphology, particle size, diffraction pattern, thermal stability, optical and recombination of photo-generated charge carrier properties were determined. The production of nanocomposites and their efficient photocatalytic capabilities were observed. The mechanism of photocatalysis involved with PC, CZP and CTP nanocomposites are well presented by suitable diagrams representing the exchange of electrons and protons among themselves with supported equations. We discovered that increasing the number of nanocomposites in the membranes boosted both photocatalytic activity and degradation rate. CdS-Zinc-PANI (CZP) and CdS-TiO_2_-PANI(CTP) nanocomposites show entrapment at the surface defects of Zinc and TiO_2_ nanoparticles due to the demolition of unfavorable electron kinetics, and by reducing the charge recombination, greater photocatalytic activity than CdS-PANI (CP) with the same nanoparticle loading was achieved. With repeated use, the photocatalysts’ efficiency dropped very little, hinting that they may be used to remove organic pollutants from water. The photocatalytic activity of CZP and CTP photocatalytic membranes was greater when compared to CdS-PANI, which may be due to the good compatibility between CdS and Zinc and TiO_2_, as well efficient charge carrier separation. PANI can also increase the split-up of photo-excited charge carriers and extend the absorption zone when combined with these nanoparticles. As a result, the development of outrageous performance photocatalysts and their potential uses in ecological purification and solar power conversion has been facilitated. The novelty of this article is to present the degradation of AB-29 Dye using nanocomposites with polymers and study the enhanced degradation rate. Few studies have been carried out on polymer nanocomposites and their application in the degradation of AB-29 dyes and remediation of water purposes. Nanoparticle CdS is a very effective photocatalyst, commonly used for water purification along with nanoparticle ZnS and TiO_2_; but cadmium ion-leaching makes it ineffective for practical and commercial use. In the present work, we have reduced the leaching of hazardous cadmium ions by trapping them in a polyaniline matrix, hence making it suitable for commercial use. We have embedded ZnS and TiO_2_ along with CdS in a polyaniline matrix and compared their photocatalytic activity, stability, and reusability, proving our nano-composites suitable for commercial purposes with enhanced activities and stabilities, which is a novelty. All synthesized nanocomposites are active within the near-ultraviolet to deep infrared (i.e., 340–850 nm). This gives us full efficiency of the photocatalysts in the sunlight and further proves the commercial utility of our nanocomposites.

## 1. Introduction

Sludge, urban run-off, agriculture wastewater, industrial wastewater treatment plants, food processing, pulp and paper manufacturing, and aquaculture all contribute to organic pollution. Pesticides, fertilizer, solvents, phenolic compounds, plasticizers, polycyclic aromatic hydrocarbons, cleansers, lubricants, heavy oils, pharmaceuticals, enzymes, and polysaccharides are examples of organic pollutants that are extremely toxic to both the environment and living things. Among all these organic pollutants, organic dyes are considered the chief hazardous pollutant to the environment and living things because they can block sunlight from entering the water stream, limiting photosynthetic reactions, and some forms of organic dyes are extremely poisonous and even cancer-causing. These dyes are increasingly employed in the leather, plating, fiber and textile, papermaking, plastics, and lithography sectors [1]. Furthermore, they color the wastewater, causing aesthetic concerns. Dyes have a complex aromatic structure that makes them more stable and difficult to remove from effluents [2]. However, if dyestuffs-containing effluents are not properly treated, they can pose serious environmental problems. As a result, the removal of organic dyes is critical for alleviating the water difficulties that the environment and humans confront [3,4,5]. Acid dye 29, an anionic dye, is commonly used in the fiber and textile industry for dyeing all-natural fibers, silk and synthetics, which are utilized to a lesser extent in several applications such as paints, plastics, and leather. However, due to the lack of confirming animal or human evidence, Acid dye has not been categorized as “toxic by consumption”. However, following intake, it may harm an individual’s health, particularly if they have pre-existing organ damage (e.g., liver, kidney), gastrointestinal tract pain, redness in the eyes, or skin irritation concerns [6].

Chemical, biological, and physical treatment methods for eliminating contaminants from wastewater have been developed and are now widely used [7]. Adsorption is one of these approaches that has received a lot of attention due to its small cost and great efficiency, as well as its ease of use [8,9,10,11,12,13,14,15,16]. As an adsorbent, organic and inorganic composite materials can promote synergistic adsorption and broaden the application [17,18]. Previous research has shown that both photocatalytic degradation and photocatalytic hydrogen production can be carried out with semiconductor photocatalysts, which can overcome many issues. If pollution oxidation and oxygen reduction do not occur concurrently, the extent of electron and hole recombination increases due to an upsurge in electrons in the conduction band. As a result, preventing electron build-up is critical for efficient photocatalytic oxidation. These redox processes have formed the necessary part of the photocatalytic mechanism [19]. These semiconductors have been shown to use UV light, accounting for just 3–5% of the solar range, which is responsible for their drawback and commercial applicability [20]. The photocatalytic activity could be improved with the manipulation of the semiconductor’s bandgap [21], which extends the light absorption of semiconductor materials to visible areas. As a result, there has been a surge in interest in the development of semiconductor materials that emit visible light. The numerous strategies for developing visible light-responsive semiconductor materials include noble metals doping, non-metallic doping, and metal/non-metal co-doping [22,23,24]. 

Many in-organic polymers nanocomposites comprising various groupings of two or more components have gained increased consideration in current research due to their intriguing physical properties and prospective uses [25,26,27]. The polymer governing the nucleation of nanoparticles may cause their growth to be inhibited by embedding or encapsulating them in the polymer [28]. It also helps with the film’s casting and particle dimension dispersal. Due to the fast separation of charges by inducing the light and considerably delayed recombination of charges, materials with delocalized conjugated structures have been extensively studied in this field [29]. The use of conductive polymers (such as polythiophene, polyaniline, and polypyrrole) to improve the performance of composite photocatalysts for photocatalytic hydrogen production, antibacterial, and photocatalytic destruction of dangerous substances is reviewed. In composite photocatalysts, conductive polymers can reduce photogenerated electron-hole pair recombination by matching band topologies with other inorganic semiconductors [30]. As a result, conducting polymers with extended-conjugated structures, as well as their derivatives, have attracted a lot of attention as photocatalytic absorbers with the potential to improve semiconductor photo-response. PANI, in particular, is a good photosensitizer that can change TiO_2_ photocatalytic properties [31].

Several papers have been published that describe the fabrication of photoactive polyaniline (PANI) nanocomposites with semiconductors such as PANI/TiO_2_ [32], PANI/BiVO_4_ [33], PANI/SiO_2_ [34], PANI/SnO_2_ [35], PANI/CdS [36], PANI/V_2_O_5_ [37] and PANI/Fe_3_O_4_/SiO_2_/TiO_2_ [38]. Anion-type pollutants, such as phosphate ions and anionic azo dyes, have also been created and demonstrated to be excellent adsorbents with PANI/TiO_2_ composites, and there is a plethora of research based on PANI’S adsorption capability and good stability with semiconductors in terms of dye degradation [39]. However, because polyaniline is a *p*-type semiconductor and CdS nanoparticles are *n*-type, combining the two will result in effective photoconductive and photocatalysis nanocomposites [40]. The semiconductor nanomaterials PbS, ZnS, ZnSe, and CdSe, belonging to the elemental group II-VI, were intensively explored due to their size-dependent optical characteristics [41,42,43,44]. Titanium (TiO_2_) nanoparticles are intriguing due to their unique physical and chemical properties, which are widely used in the applications of coatings, solar cells, and photocatalysts [45]. Due to their large band gaps, TiO_2_ and ZnS are effective photocatalysts; however, it could be activated by UV light, which accounts for solar radiations just about 4–6%; PANI–TiO_2_ composites have a narrower bandgap than pure TiO_2_ nanoparticles, which allows PANI–TiO_2_ to absorb more photons and hence, improved the photocatalytic efficiency of TiO_2_ in sunlight [46]. PANI/TiO_2_ nanocomposites have exceptional characteristics as they are the donors and acceptors of the electron [47,48,49,50,51]. CdS-TiO_2_ and CdS-ZnS sandwich-type nanocomposites were created by combining CdS with TiO_2_ and ZnS to boost the photocatalytic activity of CdS, after a few repeating cycles, which could be related to photo-corrosion of CdS. All of the photocatalytic membranes showed a minor decrease in photocatalytic activity when used often [52,53].

In the present study, CdS-ZnS and CdS-TiO_2_ were deposited on the PANI substrate to prevent Cd^2+^ ion leaching. PANI was used to examine the effects of CdS, CdS-ZnS, and CdS-TiO_2_ nanoparticles on the fundamental properties of the synthesized materials. The outcome of this study will add to our understanding of how to make conducting polymer/semiconductor nanocomposites with desirable nanostructures for use as high-performance photocatalysts in environmental purification and solar energy conversion.

## 2. Methodology

### 2.1. Materials

The materials used were Aniline, Chloroform, Sulphuric Acid (H_2_SO_4_), Ammonium Persulphate (APS), Cadmium Nitrate (Cd(NO_3_)_2_), Methanol, Zinc chloride (ZnCl_2_) and Titanium Tetra Isopropoxide (TTIP). All the chemicals were analytical grade and were utilized exactly as they were given to us.

### 2.2. Synthesis of PANI/CdS-PANI/CdS-ZnS-PANI/CdS-TiO_2_-PANI Nano Composites

PANI was created via a chemical technique that involved oxidizing aniline in H_2_SO_4_ with the aid of APS (Ammonium Persulphate). The color of the solution changed from pale to blue-green and turned to dark green by adding ammonium persulphate to it, indicating that polymerization of aniline into polyaniline occurs swiftly. The operations were performed at low temperatures for the delayed process of polymerization to create the particles in nanosize. The following steps were used to make PANI and its different nanocomposites.

### 2.3. Preparation of Polyaniline (PANI)

In 100 mL chloroform, a 0.2 M aniline solution was produced (2.462 M). In 100 mL H_2_SO_4_, another APS (0.05 M) solution was produced (1 M) and progressively mixed with the initial solution to polymerize overnight at 4–5 °C.

### 2.4. Preparation of CdS-PANI Nanocomposite (PC)

PC was made by using a single pot chemical precipitation process to in situ polymerize PANI in CdS nanoparticles. With vigorous stirring, a 100 mL aqueous solution of Cd(NO_3_)_2_ (0.085 M) was made and mixed with 50 mL methanol (24.44 M). A 100 mL aniline (0.2 M) solution produced in chloroform (2.462 M) was further mixed with this and stirred for another 60 min. The process was performed for 1 min in an H_2_S environment with vigorous stirring and then continued for another 2 h. In a separate vessel, a 0.05 M APS solution was produced in 100 mL H_2_SO_4_ (1 M). This solution was progressively added to the preceding one and left to polymerize overnight at 4–5 °C. The finished product was a dark green color.

### 2.5. Preparation of CdS-ZnS-PANI Nanocomposite (CZP)

With continuous stirring, 50 mL methanol (24.44 M) was dropped into 100 mL aqueous ZnCl_2_ (0.15 M). The stirring process was performed for 1 min in an H_2_S environment and then followed for about another 2 h, resulting in the change in color to milky white. In a separate beaker, 100 mL aqueous Cd(NO_3_)_2_ (0.085 M) was added dropwise to 50 mL methanol (24.44 M) using a magnetic stirrer. The process was continued by stirring for 1 min in an H_2_S environment and then followed for another 2 h. The color of the solution changed from clear to bright yellow. The two solutions were mixed vigorously for 2 h with vigorous stirring. The solution that resulted was yellow. Next, 100 mL aniline (0.2 M) solution produced in chloroform (2.462 M) was mixed with this reaction mixture and carried out for another 1 h using the stirring method. In a separate vessel, a 0.05 M APS solution was produced in 100 mL H_2_SO_4_ (1 M). This solution was progressively added to the preceding one and left to polymerize overnight at 4–5 °C. The finished product was a dark green color.

### 2.6. Preparation of CdS-TiO_2_-PANI Nanocomposite (CTP)

In a typical synthesis, 100 mL aqueous Cd (NO_3_)_2_ (0.085 M) was added dropwise with continuous stirring, followed by 50 mL methanol (24.44 M) for 1 min in an H_2_S environment using a magnetic stirrer and then followed the same for another 2 h. The color of the solution changed from clear to yellow. Then, 3.53 mL TTIP (Titanium Tetra Isopropoxide) (0.1 M) was mixed in the dropwise manner to this solution (20 drops per minute), followed by 2 h of stirring. The hue of the solution changed to a faint yellow. Then, 100 mL aniline (0.2 M) solution produced in chloroform (2.462 M) was dropped into this reaction mixture and stirred for 1 h. In a separate vessel, a 0.05 M APS solution was produced in 100 mL H_2_SO_4_ (1 M). This solution was progressively added to the preceding one and left to polymerize overnight at 4–5 °C. The finished product was a dark green color. Before drying in the air, all the precipitates were washed numerous times with water and acetone.

### 2.7. Characterization Techniques

Energy-dispersive X-ray spectroscopy (EDS, JEOL, JSM6510LV, Tokyo, Japan) and Fourier Transform Infrared Spectroscopy were used to examine element analysis (Spectrum 2, PerkinElmer, Waltham, MA, USA). Scanning Electron Microscopy (JEOL, JSM6510LV, Tokyo, Japan) and Transmission Electron Microscopy are used to characterize surface morphology (JEOL, JEM2100, Tokyo, Japan). Powder X-ray diffraction (Miniflex-TM II Benchtop, Rigaku Co-operation, Tokyo, Japan) was used to examine the structural properties. Thermal Gravimetric Analysis was used to determine thermal properties (TGA). UV-Visible Spectroscopy was used to determine the optical properties (Shimadzu UV-1601, Waltham, MA, USA).

### 2.8. Photocatalytic Experiment

PANI and its nanocomposites (PC, CZP, and CTP) were used in photocatalytic tests to decolorize the dye derivative Acid Blue-29 (AB-29) in visible light. The inner and outer jackets of a standard immersion well photoreactor were employed. A halogen liner lamp was used for irradiation (500 W, 9500 Lumens). In the photocatalytic experiment, 180 mL of the dye solution with the concentration of 0.06 mM and the optimized catalyst dosage (1 gL^−1^) was stirred in the shady for at least 20 min in the presence of atmospheric oxygen to achieve adsorption–desorption equilibrium between dye and catalyst surface. The initial phase (5 mL) of the solution (0 min) was then removed, and irradiation began. During the irradiation, further sections of samples (5 mL each) were collected at regular intervals and evaluated after centrifugation. Changes in absorption were used to track the decolorization of AB-29 using a UV-vis spectroscopic analysis approach (Shimadzu UV-Vis 1601). The dye concentration was determined using a standard calibration curve based on the dye’s absorbance at various known values. It is critical to assess the catalyst’s stability and reuse it to put it into practice. The photocatalytic capabilities of the nanomaterials were investigated for five consecutive cycles under similar conditions, using the same amount of catalyst nanomaterials and a fresh solution of dye sample each time.

## 3. Results and Discussion:

### 3.1. Fourier Transform Infrared (FTIR) Spectroscopy

**Figure 1:**
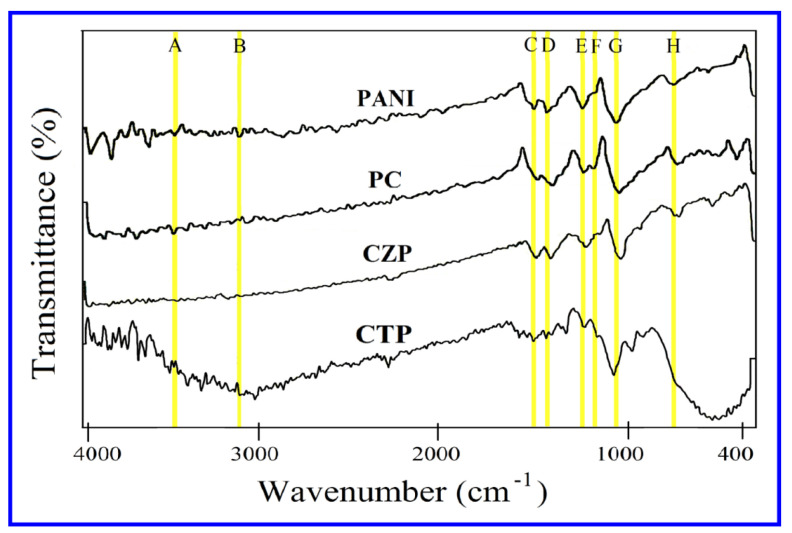
FTIR spectra of PANI. PC, CZP and CTP nanocomposites.

The FTIR analysis was used to determine the vibrational modes in PANI and its composite nanomaterials (PC, CZP, and CTP), as well as to investigate the interaction of capping molecules with the surfaces of conducting polymers and nanoparticles. Figure 1 depicts the FTIR spectra of polyaniline and its nanocomposites from a scale of 4000 cm^−1^ to 400 cm^−1^. The bands at 3460 cm^−1^ and 3230 cm^−1^ correspond to free (non-hydrogen bonded) NH stretching vibrations and hydrogen-bonded NH bonds between amine and imine sites, respectively [54,55]. The C=C bond stretching vibration peak in the quinone, and benzene ring was at 1572 cm^−1^ and 1497 cm^−1^, respectively [56], the C-N bond stretching vibration peak in the quinone ring and benzene ring was at 1301 cm^−1^, and 1245 cm^−1^, respectively [57]. Peaks observed in the 1000–1200 cm^−1^ range were caused by aromatic in-plane C-H bending modes [58]. The band at 1173 cm^−1^ was thought to be strong and was used to determine the electron delocalization, so it is a PANI conductivity characteristic peak [55]. C-H deformations in the 1,4-disubstituted benzene ring were discovered at 870 cm^−1^ [59]. The FTIR spectra of synthesized PANI matched previous findings [60].

The characteristic peak for CdS in the FTIR spectra for PC was 405 cm^−1^ [61,62]. Some PANI peaks shifted as a result of the addition of CdS nanoparticles. When compared to the pure PANI peak at 3460 cm^−1^, the band corresponding to polyaniline at 3506 cm^−1^ shifted blue, and for pure PANI peak at 1561 cm^−1^, 1489 cm^−1^, 1298 cm^−1^, 1245 cm^−1^, 1126 cm^−1^, and 813 cm^−1^ shifted red and progressively moved to 1548 cm^−1^, 1451 cm^−1^, 1290 cm^−1^, 1243 cm^−1^, 1119 cm^−1^, and 784 cm^−1^. The appearance of the band at 1119 cm^−1^ in the nanocomposite indicated the production of polyaniline [55,56,57,58]. CdS and Polyaniline established a coordination bond in PC, resulting in weaker bonds and vibrations and the conjugated system of PANI. This resulted in a higher density of electrons in CdS and, as a result, a higher number of photoelectrons, resulting in greater photocatalytic activity of nanocomposites [54].In addition, CZP has redshift at 1555 cm^−1^, 1475 cm^−1^, 1287 cm^−1^, 1227 cm^−1^, 1108 cm^−1^, and 807 cm^−1^, attributing to pure Polyaniline, representing reduced conjugating system and bond strengths of polyaniline, as well as enhanced electron density in CdS, indicating higher photocatalytic activity of CZP. The absorption bands at 405 cm^−1^ and 612 cm^−1^ showed the existence of CdS [52,62] and ZnS [63,64] in CZP, correspondingly. The Ti-O and O-Ti-O bonds in TiO_2_ were responsible for the broad CTP peaks around 400–800 cm^−1^ [55,60]. This broad peak coincided with PANI’s high of 813 cm^−1^ and CdS typical peak of 405 cm^−1^. The characteristic bending mode of water molecules adsorbed on TiO_2_ surface caused the band intensity at 1629 cm^−1^ in CTP nanocomposite [60]. The existence of a peak at 1139 cm^−1^ in CTP [55] confirmed the development of PANI. Some PANI peaks shifted due to the inclusion of TiO_2_ particles. The peaks at 3426 cm^−1^, 3079 cm^−1^, 1560 cm^−1^, 1486 cm^−1^, 1290 cm^−1^, and 1238 cm^−1^, displaced to lower wavenumbers (redshift), were weak after coupling with TiO_2_ [60]. As a result, the density of electrons in TiO_2_ rose, and the photocatalytic activity of CTP improved.

### 3.2. Energy Dispersive X-ray Spectroscopy (EDS)

**Figure 2:**
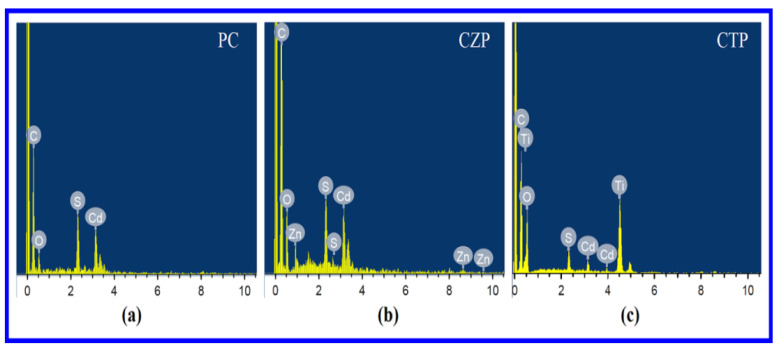
EDS spectra of (**a**) PC, (**b**) CZP and (**c**) CTP.

The EDS spectra of PC, CZP and CTP nanocomposites are shown in Figure 2. The graphs revealed the presence of carbon, oxygen, sulfur, zinc, titanium, and cadmium, which was expected. Peaks of CdS in PC, CdS and ZnS in CZP, and CdS and TiO_2_ in CTP all demonstrate that necessary semiconductors are present in Polyaniline.

### 3.3. Scanning Electron Microscopy (SEM)

**Figure 3:**
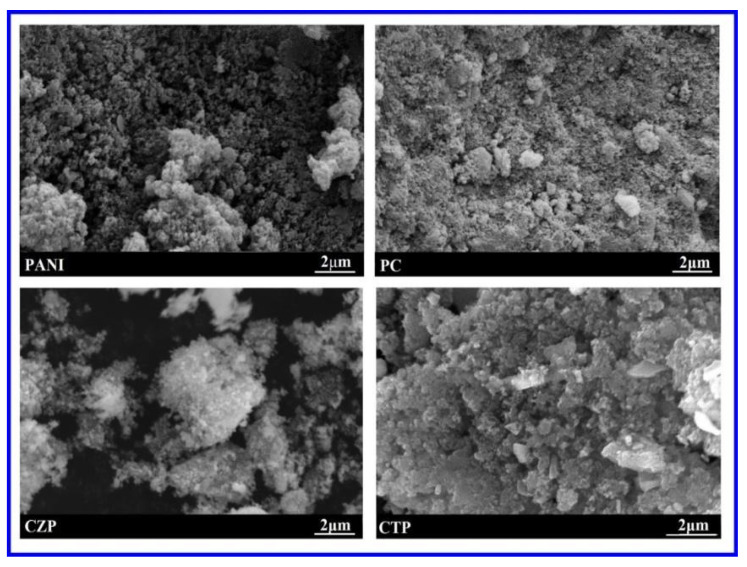
SEM images of PANI, PC, CZP and CTP nanocomposites.

Surface morphologies of the PANI, PC, CZP, and CTP nanocomposites are displayed in Figure 3. According to the SEM pictures, the catalysts contain a combination of agglomerated and non-agglomerated fluffy particles, which is consistent with previous findings [54]. In CTP nanocomposites, some surface-residing nanoparticles can be visible, but they are not evenly dispersed. From the SEM images, no apparent pores were seen on the membranes’ surface. The sub-layers of PANI, PC, CZP, and CTP appear to be slightly porous, while the top layer appears to be fully compact. When compared to the PANI, which has a few large pores, CTP and PC contain a lot of small pores.

### 3.4. Transmission Electron Microscopy

**Figure 4:**
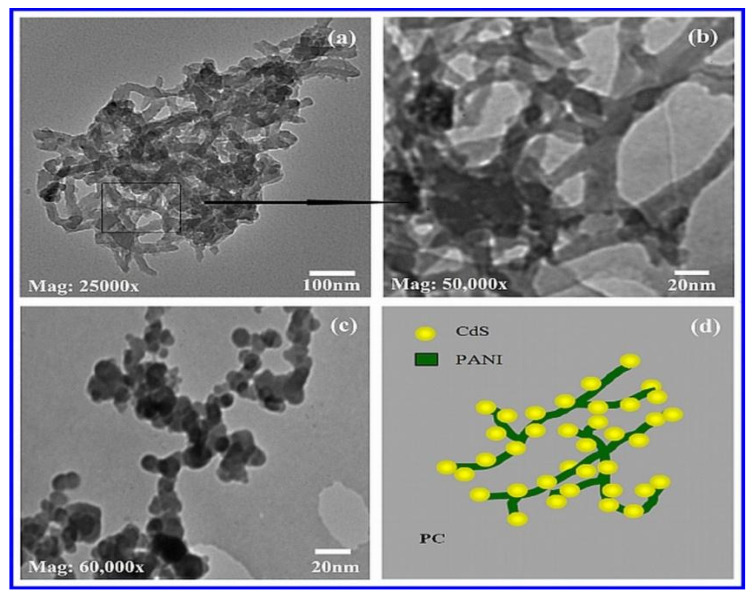
TEM images of (**a**) PANI at 25,000 times magnification, (**b**) PANI at 50,000 times magnification, (**c**) PC at 60,000 times magnification and (**d**) representative diagram of PC.

TEM images of synthesized polyaniline nanostructures are shown in Figure 4a,b (PANI). It was possible to see a huge number of interconnected branched polyaniline nanofibers. Most fibers had diameters of less than 50 nm. TEM images of PC are shown in Figure 4c. On the PANI nanofibers, homogenous spherical CdS nanoparticles were found to be well disseminated. The presence of CdS in PANI had previously been established using FTIR and EDS. It was discovered that the presence of PANI in the composites reduced the aggregation of CdS particles to some extent. As previously stated for PC, this allowed for simple electron transport from PANI to CdS, as well as easily activated photoelectrons in CdS, resulting in increased photocatalytic efficiency for dye degradation of PC. Figure 4d shows a representative diagram of a PC.

**Figure 5:**
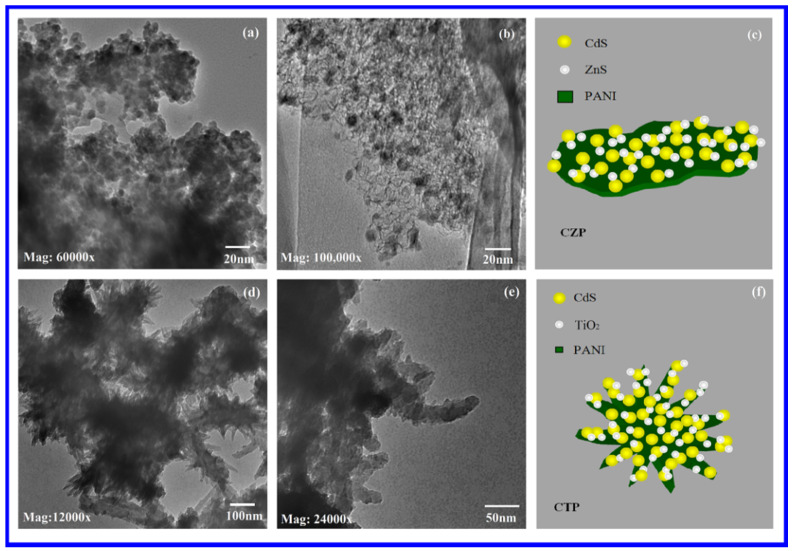
TEM images of (**a**,**b**) CZP at 60,000- and 100,000-times magnification, respectively, (**d**,**e**) CTP at 12,000- and 24,000-times magnification, respectively and (**c**,**f**) representative diagrams of CZP and CTP, respectively.

The two distinct-sized spherical nanoparticles distributed in the PANI matrix were observed and revealed by TEM images of CZP (Figure 5a,b). As demonstrated by EDS and FTIR, the generation of CdS and ZnS Sandwich type (SW-type) quantum dots (QDs) was confirmed by two different sizes and spherical structures. CdS and ZnS were found close with one another in the polymer matrix, implying the strongest bonding connecting each other, as demonstrated by FTIR. Figure 5 shows a sample schematic of CZP (c). Similarly, CTP TEM images (d) and (e) indicated two different sized CdS and TiO_2_ QDs on the surface of the PANI matrix. Figure 5 depicts a sample diagram of CTP (f). Two different-sized spherical nanostructures appeared to be nearest, as suggested by EDS and FTIR, supporting the production of CdS and TiO_2_ SW-type NCs in the polyaniline matrix with significant bonding among each other. A typical schematic of CTP is shown in Figure 5f.

### 3.5. X-ray Diffraction (XRD) Spectroscopy

**Figure 6:**
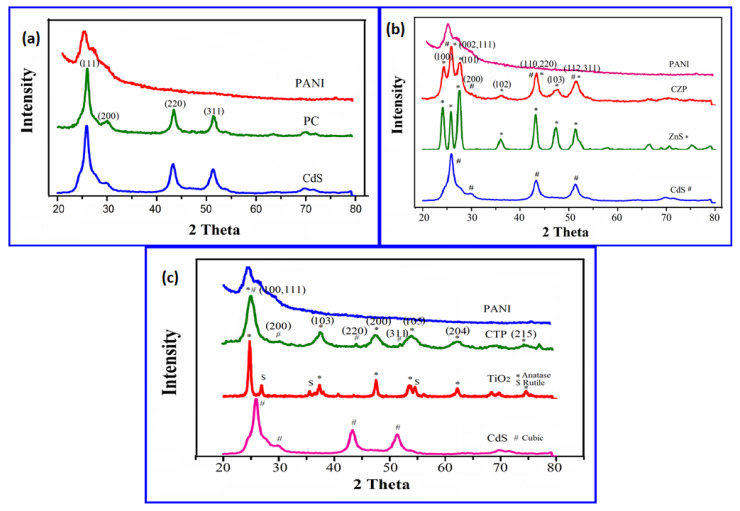
(**a**) XRD pattern of PC in comparison to cubic CdS and synthesized PANI (**b**) XRD pattern of CZP in comparison to cubic CdS, hexagonal ZnS and PANI (**c**) XRD pattern of CTP in comparison to cubic CdS, Degussa P-25 TiO_2_ and PANI.

Figure 6a shows the powder XRD patterns of PANI, PC and CdS. The characteristic broad peak of amorphous PANI centered at 2θ = 25° was observed in the figure [65]. The production of cubic CdS QDs embedded in PANI was suggested by the diffraction patterns corresponding to PC, which had peaks comparable to cubic CdS centered at 2θ = 25.955° (111), 29.428° (200), 43.372° (220), and 51.395° (311) [66]. The XRD peaks for PC were a little wider than expected, which could be attributed to the inclusion of PANI. The Scherrer formula [67,68] was used to compute the predicted crystal size of CdS, which came out to be 6.4 nm. Figure 6b shows the XRD pattern of CZP. The XRD pattern is centered at 2θ = 24.471°, 26.018°, 27.740°, 36.093°, 43.458°, 47.645° and 51.507° belonging to hexagonal ZnS (2θ = 24 (100), 26 (002), 27 (101), 36 (102), 43 (110), 47 (103) and 51 (112)) [69,70] and cubic CdS with characteristic peaks at 2θ = 26° (111), 29° (200), 43° (220), and 51° (311) [36,54,66] The XRD spectra revealed significant widening due to PANI. The crystal size of CZP was impossible to quantify due to the overlap of the peaks respective to the nanocomposites used for the catalysis process. Figure 6c shows the XRD spectrum of CTP. At 2θ = 25°, a broad peak was discovered, as the combination of anatase TiO_2_, cubic CdS, and polyaniline. The Peaks at 2θ = 37.8° (103), 48.07° (200), 54.18° (105), 62.42° (204) and 75.2° (215) corresponds to anatase TiO_2_ [71], while peaks at 29.239° (200), 43.372° (220) and 51.395° (311) were associated with cubic CdS [36,54,66]. Because of the existence of the nanocomposite peak which is used as the photocatalyst, the crystalline size was not able to deduct.

### 3.6. Thermal Analysis

**Figure 7:**
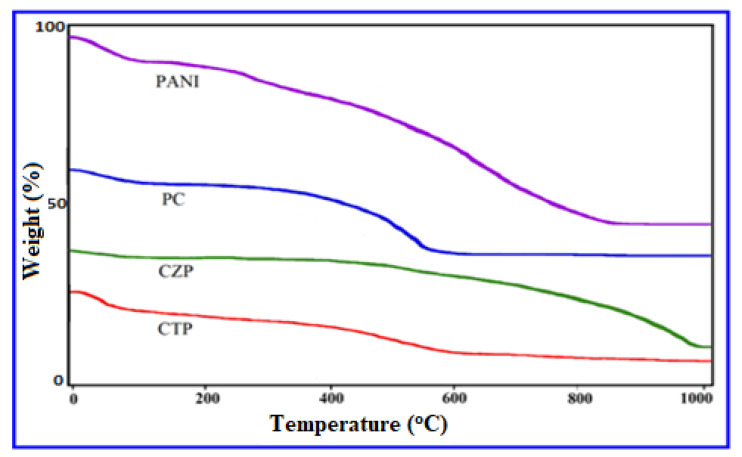
TGA curves of PANI, and its composite nanomaterials (PC, CZP, and CTP).

The decrease in weight was seen in the TGA thermograph of PANI at two temperatures in the range from 30 to 100 °C as shown in Figure 7. The sample’s first mass loss began at 20 °C and rapidly ended at 100 °C. The evaporation of absorbed moisture, the removal of contaminants, residual water, and unreacted monomers all contribute to this effect, which can be seen in a variety of polymers. The polymer breakdown started at around 210 °C (the most commonly quoted was around 300 °C) [36,65]. At 800 °C, the degradation was quite moderate, and the observed mass loss was not significant, since it did not surpass 46%. The heat resistance of the synthesized polyaniline was found to be above average in this study.

The stability was increased by the decomposition at high temperature for the sample in the TGA profile [56] compared to pure PANI (280–300 °C) and consistent thermal stability from 600 °C to 1000 °C. It could be related to the use of CdS nanoparticles in the polymer matrix, which resulted in a higher binding force due to CdS nanoparticle interaction with the lone pair electrons of the N atom in the polymer backbone. The primary weight loss peak in CZP was discovered at a significantly elevated temperature (400 °C) (which begins at about 280–300 °C) and persisted for the composites up to 980 °C, demonstrating greater composite stability due to inorganic semiconductor nanoparticles [56].TGA curve of CTP’s also revealed a rise in the second-stage breakdown temperature to 420 °C, as well as consistent thermal stability between 600 °C and 1000 °C. At 20–80 °C, the sample began to lose mass due to moisture, contaminants, and unreacted monomers. The presence of a significant association between PANI and CdS-TiO_2_ could explain the CTP nanocomposite’s increased thermal stability when compared to pure PANI.

### 3.7. Optical Analysis

**Figure 8:**
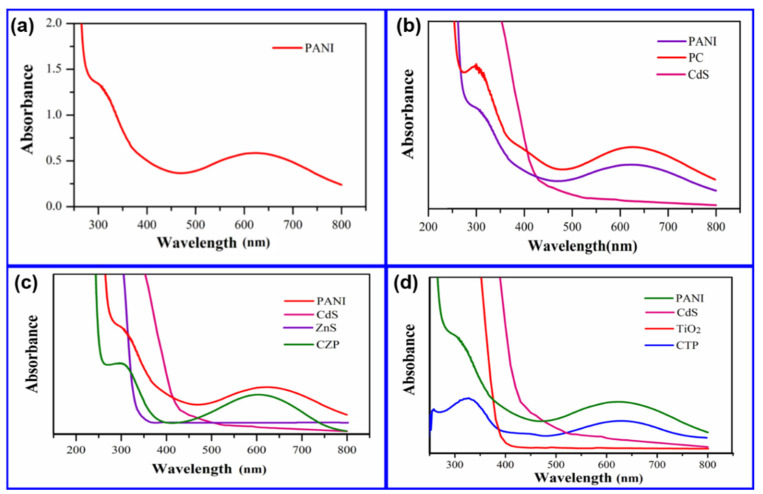
(**a**) UV-visible spectra of PANI in the range 200 to 800 nm (**b**) UV-visible spectra of PC in comparison to bulk CdS and synthesized PANI. (**c**) UV-visible spectra of CZP in comparison to bulk CdS and ZnS and synthesized PANI. (**d**) UV-visible spectra of CTP in comparison to synthesized PANI, bulk CdS and ZnS.

Figure 8a–d shows the UV-visible absorption spectra of PANI, PC, CZP, and CTP nanocomposites, respectively. The optical characteristics of the sample in N-methyl pyrrolidinone (NMP) solution were measured using a UV-Vis Spectrometer. NMP is a non-protonating polyaniline solvent that is extremely rare [65]. The absorption spectra of PANI (Figure 8) revealed two absorption peaks at 320 nm and 630 nm, which correspond to the polyaniline emeraldine base form. The excitation of benzenoid ring was attributed to the intensity band at 320 nm, while the absorption at 630 nm was attributed to a benzenoid to quinoid excitonic transition [72,73]. The existence of typical peaks of π-π* transitions (at λ = 420–450 nm and 760–800 nm) without any indications of transitions involving polarons showed that the prepared polyaniline was due to the emeraldine base form of polyaniline [65]. Figure 8b depicts the absorption spectra of PC. PC had two prominent peaks at 320 nm and 630 nm, with one hump at 420 nm, in its absorption spectra. The π-π* transition in the benzenoid ring and the benzenoid to quinoid excitonic transition of polyaniline were attributed to the peaks at 320 nm and 630 nm, respectively, indicating emeraldine base type polyaniline. CdS QDs were responsible for the shoulder at 420 nm, which displayed a significant blue shift from the bulk (510 nm) [74]. As a result, there appears to be a substantial interaction between CdS and PANI. Figure 8c depicts the absorption spectrum of CZP. The spectrum indicated just two peaks centered at 340 and 620 nm, with the strength of the spectrum increasing compared to pure PANI. Some authors [75] report a rise in the peak intensity corresponding to polyaniline in addition to the semiconductor nanocomposites. The appearance of band intensity at 340 and 620 nm has corresponded to the emeraldine base type of the PANI matrix. Instead of the peaks attributing to CdS (512 nm) [74] and ZnS (336 nm) [76], the peak at 340 nm displayed broadening from 280 to 350 nm with a tail in the visible region up to 400 nm, demonstrating the strong bonding of CdS and ZnS nanoparticles with polyaniline. PANI’s absorption spectrum revealed a blue shift in the presence of the CdS-ZnS nanocomposite without revealing any extra peaks, and the augmentation in the absorption spectrum was analyzed because of the significant interaction [59,75]. An improved photoresponse was predicted in comparison to pure PANI and CdS-ZnS sandwich nanocomposite. Figure 8d depicts the absorption spectrum of CTP. CTP’s absorption spectrum revealed two large peaks at 330 nm and 640 nm, as well as a faint broad peak at 450 nm. The absorption intensity of the CTP spectra was reduced when compared to pure PANI. CdS QDs were responsible for the faint peak at 450 nm, while emerald base polyaniline was responsible for the peaks at 330 and 640 nm. Colloidal TiO_2_ nanoparticles’ absorption overlapped with polyaniline, which caused the peak at 330 nm to grow dramatically from 290 to 350 nm, with a tail in the visible region up to 400 nm. The blue shift and decrease in intensity of the CTP revealed a significant interaction of quantum dots (CdS and TiO_2_) and PANI [59]. CTP may be activated through the absorption of UV and visible light (between 190 and 800 nm), which is an excellent choice for photocatalytic applications.

### 3.8. Photocatalytic Activity:

Acid blue dye was used to test the photocatalytic activity of CdS, CdS-ZnS, and CdS-TiO2 nanoparticles mounted on PANI membranes (AB-29).

#### 3.8.1. Photocatalysis by PC

**Figure 9:**
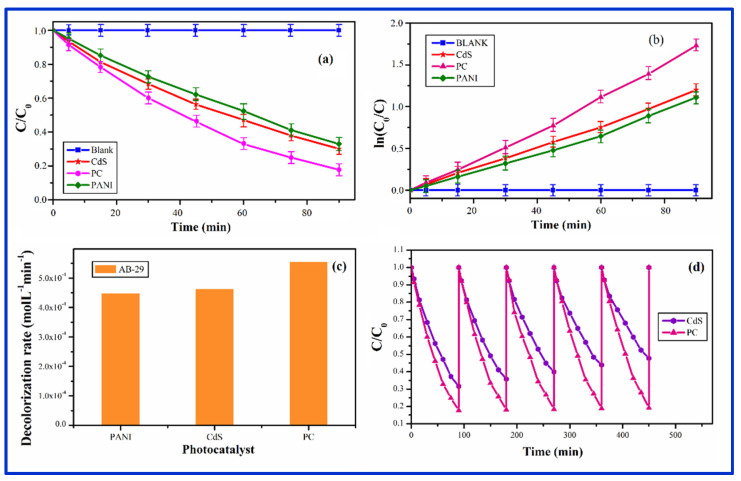
(**a**) Change in concentration of AB-29 with time in the presence and absence of PC in comparison to PANI and CdS nanoparticles. (**b**) Change in concentration of AB-29 with time in the presence and absence of PC in comparison to PANI and CdS nanoparticles. (**c**) The decolorization rate of AB-29 in the presence of synthesized PC in comparison to PANI and CdS nanoparticles. (**d**) Stability and recycle of PC in comparison to pure CdS.

The photocatalytic response of PC is depicted in Figure 9a–d. Our results showed that while PANI had only a minor effect on photocatalyst activity, it was beneficial for improving photocatalyst stability. PC demonstrated better photocatalytic degradation activity compared to pure CdS and PANI. The reasons for PC’s improved photocatalytic activity are as follows: first, the density of the photoelectrons in CdS was increased by the transfer of photoexcited electrons. Second, the separation of the charges and the charge density were increased by the attraction of all the holes from CdS by PANI. Third, the addition of CdS caused some order to the disordered structure of PANI, and PANI prevented some agglomeration and size growth of CdS particles, resulting in efficient and rapid photodegradation of adsorbed contaminants [77]. The relative change in the concentration (C/C0) of AB-29 is shown in Figure 9a with and without the catalysts (PC, PANI, and pure CdS NP). After 90 min of irradiation decolorization of AB-29 was 82.2%, 69.8%, and 67.1%, respectively, as shown in Figure 9a. In the absence of photocatalyst, however, there was no discernible decrease in dye concentration. The decolorization curve (Figure 9a) indicated a pseudo-first-order response, which was also shown in Figure 9b. The slope of ln (C_0_/C) versus t will be k_app_. For all of the studies, the correlation constant (R^2^) for the fitted lines was calculated to be around 0.99. The decolorization rate (molL^−1^min^−1^) of AB-29 was calculated using the rate constant obtained from Figure 9b. The computed rates (molL^−1^min^−1^) were 4.47 × 10^−4^ (PANI), 4.62 × 10^−4^ (CdS), and 5.54 × 10^−4^ (PC). Figure 9c depicts the decolorization rate of AB-29 breakdown of several photocatalysts (PC, PANI, and pure CdS NP). It was critical to assess the catalyst’s stability and reuse to put it into practical usage. The photodegradation of AB-29 during five successive cycles with the same 1 gL^−1^ catalyst at 0.06 mM dye concentration is shown in Figure 9d. Before each photocatalytic run, the nanocomposite catalyst was rinsed with double distilled water after each cycle and a fresh solution of AB-29 was added. After 90 min of reaction time, the relative decolorization utilizing PC for the 5-cycling reuse was 82.3%, 81.8%, 81.6%, 81.1%, and 80.8%, respectively. The catalytic activity of PC reduced little following the first cycles, whereas the activity of pure CdS decreased dramatically. The runoff from the PC while washing and filtering could be the cause of this minor movement. However, in subsequent studies, the decolorization efficiency remained rather constant, indicating that CdS QDs were consistently mixed within the PANI matrix. In this regard, the recycle of PC photocatalyst confirmed the PC’s relative stability. As a result, PC appears to be a superior photocatalyst to pure CdS and PANI, with increased activity and stability.

Electrons were scavenged by molecular oxygen O_2_ in oxygen-equilibrated environments, resulting in the superoxide radical anion O_2_·^−^. (Equation (5)) and hydrogen peroxide H_2_O_2_ (Equation (6)). These newly formed intermediates combined to form the hydroxyl radical ·OH (Equation (7)). The photo-generated hole at PANI oxidized the hydroxyl groups (OH) (Equation (8)) and the water (H_2_O) molecule (Equation (9)), resulting in hydroxyl radicals (·OH). The ·OH radical, which is a powerful oxidizing agent capable of degrading a wide range of contaminants, played a role in the degradation of the dye AB-29 (Equation (10)) [77,78].

The reactions can be expressed as follows:

(1) $$CdS/PANI\;h\nu \to \;CdS\left(e^{-}+h^{+}\right)/PANI$$

(2) $$CdS/PANI\;h\nu \to \;CdS/PANI\left(e^{-}+h^{+}\right)$$

(3) $$CdS\left(e^{-}+h^{+}\right)/PANI\to CdS\left(e^{-}\right)/PANI\left(h^{+}\right)$$

(4) $$CdS/PANI\left(e^{-}+h^{+}\right)\to CdS\left(e^{-}\right)/PANI\left(h^{+}\right)$$

(5) $$CdS\left(e^{-}\right)/PANI\left(h^{+}\right)+O_{2}\;\to \;CdS/PANI\left(h^{+}\right)+O_{2}^{·-}$$

(6) $$CdS\left(e^{-}\right)/PANI\left(h^{+}\right)+O_{2}+2H^{+}\to \;CdS/PANI\left(h^{+}\right)+H_{2}O_{2}$$

(7) $$H_{2}O_{2}+O_{2}^{·-}\;\to \;·OH+OH^{-}+O_{2}$$

(8) $$CdS\left(e^{-}\right)/PANI\left(h^{+}\right)+H_{2}O_{absorbed}↔CdS\left(e^{-}\right)/PANI+H^{+}+·OH$$

(9) $$CdS\left(e^{-}\right)/PANI\left(h^{+}\right)+OH_{absorbed}^{-}↔CdS\left(e^{-}\right)/PANI+·OH$$

(10) ·OH+dye →degradation products

#### 3.8.2. Photocatalysis by CZP

**Figure 10:**
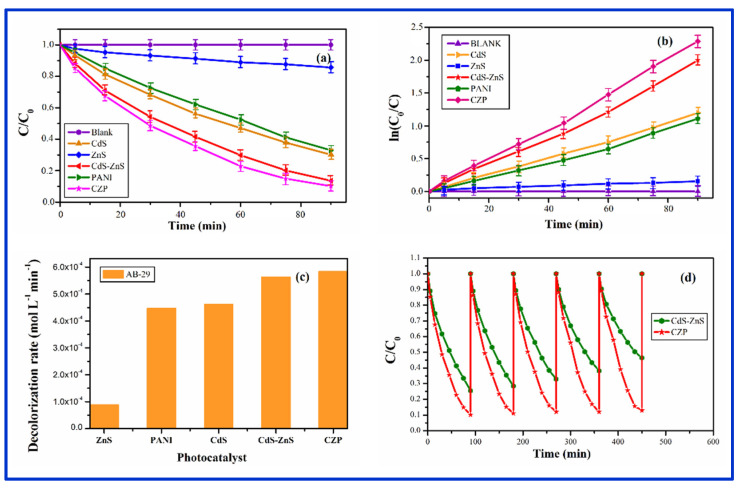
(**a**) Change in concentration of AB-29 with time in the presence and absence of CZP in comparison to PANI, CdS and ZnS nanoparticles and CdS-ZnS nanocomposite (**b**) Change in concentration of AB-29 with time in the presence and absence of CZP in comparison to PANI, CdS and ZnS nanoparticles and CdS-ZnS nanocomposite (**c**) The decolorization rate of AB-29 in the presence of synthesized CZP in comparison to PANI, CdS and ZnS nanoparticles and CdS-ZnS nanocomposite (**d**) Stability and recycle of CdS-ZnS nanocomposite embedded in PANI (CZP), in comparison to free CdS-ZnS nanocomposite without PANI.

**Figure 11:**
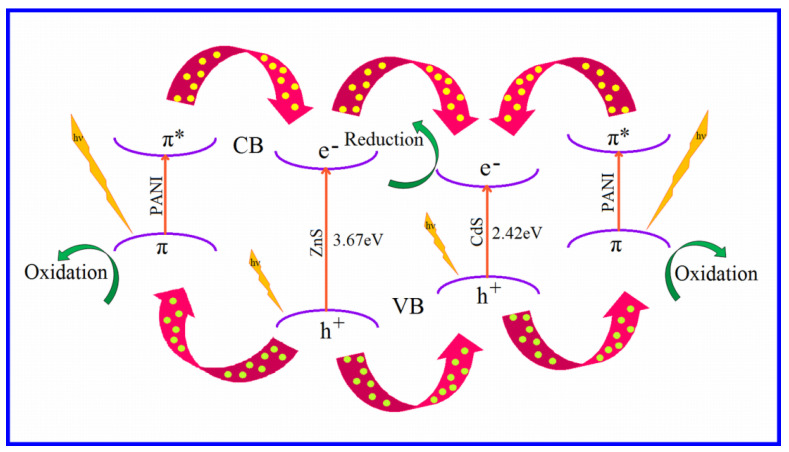
Representative diagram of the mechanism of photocatalysis by CZP.

Figure 10a–d show the photocatalytic response of CZP in comparison to pure CdS, ZnS, and PANI. CZP had higher photocatalytic degradation activity than pure CdS, ZnS, PANI, and CdS-ZnS NC. In Figure 10a, the relative change in concentration (C/C_0_) of organic dye AB-29 was plotted as a function of time with and without photocatalysts (CZP, PANI pure CdS, and ZnS QDs, and CZS-ZnS NC). When compared to pure CdS or pure PANI, CZP demonstrated a significant increase in photoactivity, but only a minor increase when compared to CdS-ZnS NC. Although PANI only slightly increased the catalytic efficiency of CdS-ZnS NC (in CZP), it was discovered to be more beneficial for improving photocatalyst stability when compared to pure CdS-ZnS NC in the absence of polyaniline, as polyaniline reduced CdS photo corrosion by attracting all holes to itself. The decolorization of AB-29 after 90 min of irradiation with CZP, pure CdS-ZnS NC, CdS QD, PANI, and ZnS QD was 89.8 percent, 86.5%, 69.8%, 67.1%, and 14.2%, as shown in Figure 10a. The decolorization curve (Figure 10a suggested a pseudo-first-order response, which was confirmed by plotting ln(C_0_/C) vs. irradiation time in Figure 10b. The correlation constant (R^2^) for the fitted lines was calculated to be around 0.99 for all the studies. The rate constant obtained from Figure 10b was used to compute the decolorization rate (molL^−1^min^−1^) of AB-29. The computed decolorization rates for ZnS, PANI, CdS, CdS-ZnS, and CZP were 0.89 × 10^−4^ < 4.47 × 10^−4^ < 4.62 × 10^−4^ < 5.63 × 10^−4^ < 5.84 × 10^−4^molL^−1^min^−1^, respectively. Figure 10c depicts the decolorization rate of AB-29 breakdown with several photocatalysts (ZnS, PANI, CdS, CdS-ZnS and CZP). Figure 10d depicts the photodegradation of AB-29 during a five-cycle period. After 90 min of reaction time, the relative decolorization utilizing CZP for the 5-cycling reuse was 89.8%, 88.9%, 88.0 percent, 87.9%, and 87.1%, respectively. The photocatalytic activity of CZP reduced slightly after the first cycles, compared to pure CdS-ZnS NC in the absence of polyaniline the catalytic activity was reduced dramatically (74.5%, 71.5%, 67.1%, 61.8% and 53.5%). The CZP runoff during washing and filtering could be the cause of this modest drop. However, in subsequent experiments, the decolorization efficiency remained rather constant. The recycling of CZP photocatalyst confirmed the relative stability of the CZP in this regard. As a result, CZP appears to be a superior photocatalyst to pure CdS-ZnS in terms of activity and stability [79]. Figure 11 depicts a schematic representation of the CZP mechanism, which involves photoexcited electrons and holes.

The following are examples of reactions:

(11) $$CdS/ZnS/PANI\;h\nu \to \;CdS\left(e^{-}+h^{+}\right)/ZnS/PANI$$

(12) $$CdS/ZnS/PANI\;h\nu \to \;CdS/ZnS/PANI\left(e^{-}+h^{+}\right)$$

(13) $$CdS/ZnS/PANI\;h\nu \to \;CdS/ZnS\left(e^{-}+h^{+}\right)/PANI$$

(14) $$CdS\left(e^{-}+h^{+}\right)/ZnS/PANI\to CdS\left(e^{-}\right)/ZnS/PANI\left(h^{+}\right)$$

(15) $$CdS/ZnS/PANI\left(e^{-}+h^{+}\right)\to CdS\left(e^{-}\right)/ZnS/PANI\left(h^{+}\right)$$

(16) $$CdS/ZnS\left(e^{-}+h^{+}\right)/PANI\to CdS\left(e^{-}\right)/ZnS/PANI\left(h^{+}\right)$$

(17) $$CdS\left(e^{-}\right)/ZnS/PANI\left(h^{+}\right)+O_{2}\;\to \;CdS/ZnS/PANI\left(h^{+}\right)+O_{2}^{·-}$$

(18) $$CdS\left(e^{-}\right)/ZnS/PANI\left(h^{+}\right)+O_{2}+2H^{+}\to \;CdS/ZnS/PANI\left(h^{+}\right)+H_{2}O_{2}$$

(19) $$H_{2}O_{2}+O_{2}^{·-}\;\to \;·OH+OH^{-}+O_{2}$$

(20) $$CdS\left(e^{-}\right)/ZnS/PANI\left(h^{+}\right)+H_{2}O_{absorbed}↔CdS\left(e^{-}\right)/ZnS/PANI+H^{+}+·OH$$

(21) $$CdS\left(e^{-}\right)/ZnS/PANI\left(h^{+}\right)+OH_{absorbed}^{-}↔CdS\left(e^{-}\right)/ZnS/PANI+·OH$$

(22) ·OH+dye →degradation products

#### 3.8.3. Photocatalysis by CTP

**Figure 12:**
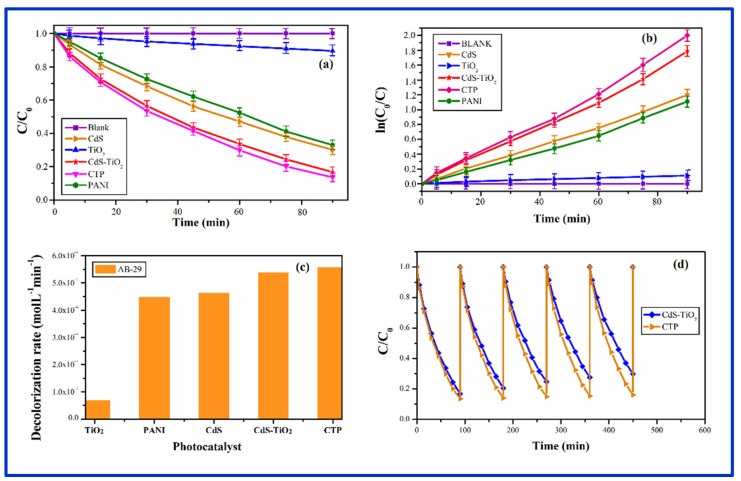
(**a**) Change in concentration of AB-29 with time in the presence and absence of CTP in comparison to PANI, CdS and TiO_2_ nanoparticles and CdS-TiO_2_ nanocomposite (**b**) Change in concentration of AB-29 with time in the presence and absence of CTP in comparison to PANI, CdS and TiO_2_ nanoparticles and CdS-TiO_2_ nanocomposite (**c**) The decolorization rate of AB-29 in the presence of synthesized CTP in comparison to PANI, CdS and TiO_2_ nanoparticles and CdS-TiO_2_ nanocomposite (**d**) Stability and recycle of CdS-TiO_2_ nanocomposite embedded in PANI (CTP) in comparison to free CdS-TiO_2_ nanocomposite without PANI.

The photocatalytic performance of CdS-TiO_2_ nanocomposites on polyaniline is shown in Figure 12a–d. CTP outperformed pure CdS, TiO_2_, PANI, and CdS-TiO_2_ in terms of photocatalytic degradation (without PANI). In comparison to CdS-TiO_2_, our findings showed that PANI had only a minor influence on CTP activity while significantly increasing CTP stability (CdS-TiO_2_ NC in the absence of Polyaniline. It was feasible to push photogenerated charges by attaching TiO_2_ and PANI to the surface of CdS, resulting in more effective charge separation in CTP [80]. Figure 12a depicts the relative change in concentration (C/C0) of the organic dye AB-29 as a function of time, with and without photocatalysts (CTP, PANI pure CdS and TiO_2_ QDs, and CdS-TiO_2_) (CTP, PANI pure CdS and TiO_2_ QDs, and CdS-TiO_2_). As shown in Figure 12a, after 90 min of irradiation with CTP, CdS-TiO_2_, CdS QD, PANI, and TiO_2_ quantum dots, the decolorization of AB-29 was 86.4%, 83.2%, 69.8%, 67.1%, and 10.4%, respectively. However, the absence of the photocatalyst does not have any discernible reduction in the concentration of the dye. According to the photocatalytic results, CTP had a significant increase in photoactivity when compared to pure CdS, TiO_2_, or pure PANI, but only a minor increase when compared to CdS-TiO_2_ NC. The decolorization curve (Figure 12a) indicated a pseudo-first-order reaction, which was also demonstrated by plotting ln (C_0_/C) versus irradiation time in Figure 12b. The correlation constant (R^2^) for the fitted lines was calculated to be around 0.99 for all of the studies. The rate constant obtained from Figure 12b was used to compute the degradation rate (molL^−1^min^−1^) of AB-29. The computed degradation rates for TiO_2_, PANI, CdS, CdS-TiO_2_, and CTP were 0.671 × 10^−4^ < 4.47 × 10^−4^ < 4.62 × 10^−4^ < 5.37 × 10^−4^ < 5.56 × 10^−4^ molL^−1^min^−1^, respectively. Figure 12c depicts the decolorization rate of AB-29 breakdown with several photocatalysts (pure TiO_2_ QDs, PANI, CdS QDs, CdS-TiO_2_ and CTP). The photodegradation of AB-29 during five consecutive cycles is depicted in Figure 12d. After 90 min of reaction time, the relative decolorization utilizing CTP for the 5-cycling reuse was 86.5%, 85.9%, 85.1%, 84.5%, and 83.8 %, respectively. Witnessing the outcome that the CTP’s catalytic activity reduced slightly after the first cycles, whereas pure CdS-TiO_2_ NC’s activity decreased dramatically (83.3%, 79.5%, 75.2%, 72.4% and 70.1%). The explanation for this drop could be due to CTP runoff during washing and filtering, which has a constant decolonization rate. The recycling of CTP photocatalyst confirmed the CTP’s relative stability in this regard, which shows that polyaniline prohibited CdS from CTP from photocorroding, hence improving its stability. As a result, it can be stated that CTP is a superior photocatalyst to pure CdS-TiO_2_ in terms of activity and stability.

**Figure 13:**
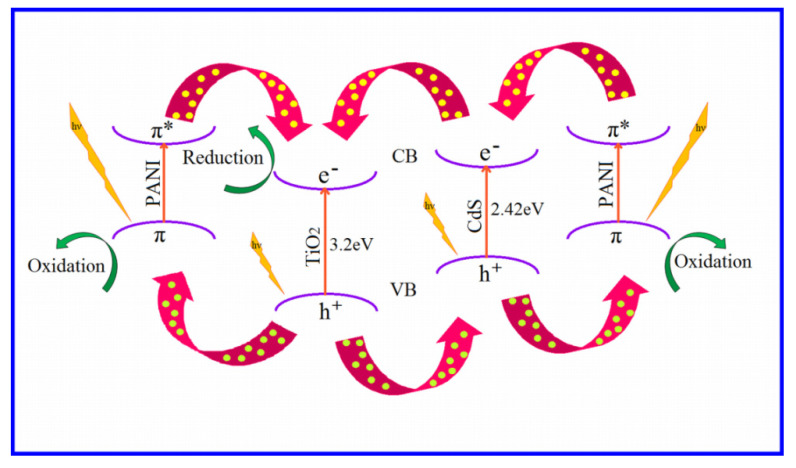
Representative diagram of the mechanism of photocatalysis by CTP.

Photo-excited electron-hole pairs were produced when CdS-TiO2-PANI nanocomposites (CTP) were bombarded with visible light, which could explain the photocatalytic response of CZP. The mechanism using photogenerated charges of CTP is depicted schematically in Figure 13.

In oxygen-equilibrated media, the electrons were scavenged by molecular oxygen O_2_, resulting in the superoxide radical anion O_2_·^−^ (Equation (29)) and hydrogen peroxide H_2_O_2_ (Equation (30)). The hydroxyl radical ·OH was produced when these newly created intermediates interacted (Equation (31)). The hydroxyl groups (OH) (Equation (32)) and the water (H_2_O) molecule (Equation (33)) were oxidized by the photo-generated hole at PANI, resulting in hydroxyl radicals (·OH). The ·OH radical behaved as a strong oxidizing agent, causing the dye AB-29 to deteriorate (Equation (34)). The process was articulated by the following equation:

(23) $$TiO_{2}/CdS/PANI\;h\nu \to \;TiO_{2}/CdS\left(e^{-}+h^{+}\right)/PANI$$

(24) $$TiO_{2}/CdS/PANI\;h\nu \to \;TiO_{2}/CdS/PANI\left(e^{-}+h^{+}\right)$$

(25) $$TiO_{2}/CdS/PANI\;h\nu \to \;TiO_{2}\left(e^{-}+h^{+}\right)/CdS/PANI$$

(26) $$TiO_{2}/CdS\left(e^{-}+h^{+}\right)/PANI\to TiO_{2}\left(e^{-}\right)/CdS/PANI\left(h^{+}\right)\;$$

(27) $$TiO_{2}/CdS/PANI\left(e^{-}+h^{+}\right)\to TiO_{2}\left(e^{-}\right)/CdS/PANI\left(h^{+}\right)$$

(28) $$TiO_{2}\left(e^{-}+h^{+}\right)/CdS/PANI\to TiO_{2}\left(e^{-}\right)/CdS/PANI\left(h^{+}\right)$$

(29) $$TiO_{2}\left(e^{-}\right)/CdS/PANI\left(h^{+}\right)+O_{2}\;\to \;TiO_{2}/CdS/PANI\left(h^{+}\right)+O_{2}^{·-}$$

(30) $$TiO_{2}\left(e^{-}\right)/CdS/PANI\left(h^{+}\right)+O_{2}+2H^{+}\to \;TiO_{2}/CdS/PANI\left(h^{+}\right)+H_{2}O_{2}$$

(31) $$H_{2}O_{2}+O_{2}^{·-}\;\to \;·OH+OH^{-}+O_{2}$$

(32) $$TiO_{2}\left(e^{-}\right)/CdS/PANI\left(h^{+}\right)+H_{2}O_{absorbed}↔TiO_{2}\left(e^{-}\right)/CdS/PANI+H^{+}+·OH$$

(33) $$TiO_{2}\left(e^{-}\right)/CdS/PANI\left(h^{+}\right)+OH_{absorbed}^{-}↔TiO_{2}\left(e^{-}\right)/CdS/PANI+·OH$$

(34) ·OH+dye →degradation products

Comparative Analysis: Table 1 represents the earlier reported work on the degradation of AB-29 dyes.

## 4. Conclusions

Semiconductor polyaniline nanocomposites were prepared using chemical precipitation method to increase the physical, chemical, optical and catalytic capabilities of semiconductors under ambient circumstances. The semiconductor nanoparticles were well incorporated into the polyaniline matrix, which increased the thermal strength of PANI’s and their optical and photocatalytic characteristics. PC stands for cubic CdS implanted on emeraldine base type polyaniline nanofibers in a CdS and PANI nanocomposite. The existence of CdS and ZnS in PANI was verified by EDX and FTIR. The frequency of the FTIR spectra was shifted to a lower value (redshift), which could be attributable to electron transfer from PANI to CdS and CdS-ZnS. XRD and UV-vis measurements both confirmed the cubic CdS and emeraldine base type Polyaniline. The thermal stability and optical properties of the PC have improved. PC also had better photocatalytic stability than pure CdS, implying that PANI decreased Cd^2+^ photo corrosion. CZP is a nanocomposite of CdS and ZnS embedded in PANI film. The microscopic examinations demonstrated that both the materials are close together in the PANI matrix, implying its development in Polyaniline. As a result, there appears to be substantial interaction between CdS-ZnS and PANI. CZP outperformed CdS-ZnS in terms of thermal stability, photocatalytic response, and stability. CTP is made up of CdS-TiO_2_ nanocomposites that have been implanted in a PANI matrix. Cubic CdS and anatase TiO_2_ were discovered in the XRD data. The presence of emeraldine base type Polyaniline was established by the UV-vis spectrum. Microscopic examinations revealed the presence of sandwich-type CdS-TiO_2_ nanoparticles embedded in the PANI matrix. FTIR, XRD, UV-vis spectra and photocatalytic reaction all revealed a significant interaction between CdS-TiO_2_ and PANI. By comparing pure with the CdS-TiO_2_ nanocomposite, the photocatalytic efficiency was found to be more stable for up to five cycles. Although PANI has only a minor contribution to the photocatalytic activity of CdS and CdS-ZnS and CdS-TiO_2_, we may conclude that it is advantageous for enhancing the stability of these nanomaterial photocatalysts. The photoactivity of the synthesized nanocomposites was found well within the visible region, hence this, along with improved stability, makes them suitable for commercial use under sunlight.

## Figures and Tables

**Table 1:** Represents the comparison results of degradation of AB-29 dyes.

S.No.	Synthesized Material or Medium	Rate of Degradation (%)	Decolorization Rate (molL^−1^min^−1^)	References
1.	Sulfuric acid-activated slug	95%		[81]
2.	Activated electric arc furnace slag	98%		[82]
3.	Zeolite/chitosan composite		The Langmuir monolayer adsorption capacities of the Z-AC/C composites are 212.76, 238.09, and 270.27 mg/g for AB29 at 30 °C, 40 14 °C, and 50 °C, respectively	[83]
4.	In the presence and absence of air by Fenton and Fenton-like processes using hydrogen peroxide (HP) and sodium persulfate (SPS), respectively, as oxidants	95.8% at pH 3 in 180 min.		[84]
5.	Montmorillonite K10-Cu(II)ethylenediamine (MMTK10-Cu(en)2)	83.27%		[85]
6.	In the presence/absence of sodium hydroxide (NaOH)	98% (in absence of NaOH)100% (in presence of NaOH)		[86]
7.	Polyaniline (PANI) with CdS (PC) PANI with CdS-ZnS (CZP) PANI with CdS-TiO2 (CTP)	82.2 89.8 89.8	5.54 × 10^−4^ 5.84 × 10^−4^ 5.56 × 10^−4^	Present Study

## Data Availability

All relevant data are within the paper.
